# Subjective Memory Ability and Long-Term Forgetting in Patients Referred for Neuropsychological Assessment

**DOI:** 10.3389/fpsyg.2016.00605

**Published:** 2016-05-02

**Authors:** Sieberen P. van der Werf, Sofie Geurts, Maartje M. E. de Werd

**Affiliations:** ^1^Brain and Cognition, Department of Psychology, University of AmsterdamAmsterdam, Netherlands; ^2^Department of Psychiatry and Medical Psychology, OLVGAmsterdam, Netherlands; ^3^Department of Medical Psychology, Canisius Wilhelmina ZiekenhuisNijmegen, Netherlands; ^4^Radboud Expert Centre for Psychology and Medicine, Department of Medical Psychology, Radboud University Nijmegen Medical CenterNijmegen, Netherlands

**Keywords:** long-term forgetting, episodic memory, neuropsychological tests, memory consolidation, memory complaints

## Abstract

It has been suggested that the memory complaints of patients who are not impaired on formal memory tests may reflect accelerated forgetting. We examined this hypothesis by comparing the 1-week delayed recall and recognition test performance of outpatients who were referred for neuropsychological assessment and who had normal memory performance during standard memory assessment with that of a non-patient control group. Both groups performed equally in verbal learning and delayed recall. However, after 1 week, the patients performed worse than controls on both recall and recognition tests. Although subjective memory ability predicted short-term memory function in patients, it did not predict long-term delayed forgetting rates in either the patients or controls. Thus, long-term delayed recall and recognition intervals provided no additional value to explain poor subjective memory ability in the absence of objective memory deficits.

## Introduction

In clinical practice, we regularly meet patients who express memory concerns and report excessive day-to-day forgetfulness while their memory test performance is well within the normal range. In clinical studies such a lack of association between subjective memory complaints and memory test performances is not uncommon (Vestberg et al., 2007; Aben et al., 2011). In some patients a lack of association might reflect disease factors such as anosognosia, while in other patients psychological factors such as stress or depression might impact the report of subject memory ability. Nevertheless, it has been suggested that the use of long-term forgetting rates may be a more sensitive method to detect subtle memory problems (Butler and Zeman, 2008). Some patients indeed argue that the ‘short’ test sessions do not reflect their problems in daily life and suggest that that their memory problems reflect increased forgetting over days instead of minutes.

Such an observation would be in agreement with the so-called “standard consolidation theory” that suggests that memories gradually become independent of the hippocampal region (Squire and Alvarez, 1995) and involves an active and ongoing process of reorganization that may continue for months or even years (McGaugh, 2000; Gold, 2006). Theoretically, an active and ongoing long term consolidation process might be more sensitive for subtle disturbing factors such as poor sleep, lower oxygen supply to the brain, subtle white matter lesions or subclinical epileptic activity, than immediate learning and recall. Several studies have indeed found that patients with memory complaints may show near-normal performances on a standard 30-min delayed recall test but may show abnormal forgetting after a prolonged period. Most evidence for this so-called accelerated forgetting has been reported for patients with temporal lobe epilepsy (Elliott et al., 2014). In other clinical patients groups, for example patients with Alzheimer disease, Multiple Sclerosis, traumatic brain injury or mild cognitive impairment, there is far less conclusive evidence for the existence of accelerating forgetting. In a recent review among a variety of patient groups we found that only three out of eleven long-term forgetting studies showed increased forgetting rates (Geurts et al., 2015). These three studies were based on comparisons of relatively small patient and control (max *N* = 15) samples (Carlesimo et al., 1995; Manes et al., 2008; Walsh et al., 2014). Only two of the 11 reviewed studies and none of the three studies that found increased long-term forgetting, combined recall and recognition scores (DeLuca et al., 1998; Gaudino et al., 2001).

Moreover, none of the reviewed studies related accelerated forgetting rates to a quantitative measure of subjective memory ability. Therefore, the main aim of this study was to test whether the differences in long-term forgetting of newly learned verbal material could explain the differences in subjective memory ability ratings in patients who were referred for neuropsychological assessment but who had normal memory performance during standard memory assessment. In addition, we compared the long term forgetting rates of the patient group to that of control group.

## Materials and Methods

### Participants and Procedure

The study procedure was evaluated and approved for the patients by the local medical ethical committee of the Radboud University Nijmegen Medical Center and approved for the healthy control subjects by the research ethical committee of the University of Amsterdam. All subjects gave written informed consent in accordance with the Declaration of Helsinki.

Our main analysis was to test for a possible association between forgetting rates and subjective memory report ratings. We considered 10% shared variance (*r* = 0.32) as a minimal effect-size to be clinically relevant. In order to get a power of 0.80 with an alpha set at 0.05 (two-sided) we had to include at least 59 outpatients. Therefore, our goal was to collect data of 60 outpatients who were referred for neuropsychological assessment and who reported cognitive problems but whose memory scores fell well within the normal range (*T*-scores of the summed score of the 5 learning trials and the *T*-scores of the recall after 30 min exceeding 35) on the Dutch version (15-word test) of the Rey Auditory Verbal Memory Test. These data were collected in three participating hospitals over a 3-year time period.

The control participants were selected from a separate study that compared long term forgetting rates to demographic variables and outcome on an experimental accelerated forgetting questionnaire. In this study, the mean age was significantly lower compared to our patient group, and there was an uneven gender distribution with significantly more females than males. Therefore we selected only participants over 30 years of age, additional care was given to equate the gender distribution in the patient group. Similar to our patient group, only participants were selected whose *T*-scores of the summed score of the 5 learning trials and the *T*-scores of the recall after 30 min exceeded 35. This selection procedure resulted in a control group of 41 participants. The demographic characteristics of this group were subsequently compared to that of the patient group.

The outpatients underwent a clinical neuropsychological assessment. Prior to this assessment the patients were asked to fill out the MMQ. After baseline assessment appointments were scheduled to discuss outcome of the clinical assessment either by telephone (*N* = 10) or at the clinic (*N* = 55). At follow-up the patients were informed about the purpose of the study and after giving informed consent, they were asked to recall the previously learned 15 words. Subsequently, the patients underwent a second recognition task. The controls underwent memory testing with the 15WT and filled out the Multimodal Memory Questionnaire (MMQ) and an experimental accelerated forgetting questionnaire. The control participants were asked consent but kept naïve about the long term memory follow up. It was agreed upon that they would be contacted by telephone to provide feedback about their baseline results. After 1 week the same testing procedure was followed as described for the outpatient group.

### Materials

The Dutch version of the RAVLT was used to assess verbal learning as well as both short-term and long-term delayed recall and recognition (Saan and Deelman, 1986). The 15WT consists of a list of 15 non-associated words that is presented orally over five trials. The total learning score is the sum of these five trials, with a possible score ranging from 0 to 75. In the original RAVLT, this initial learning phase is followed by a single presentation of a new word list to test for interference effects. In the 15WT used in this study, the interference trial was omitted. The standard testing procedure involved a 20- to 30-min delayed recall condition (30minRecall score range: 0–15) and a recognition condition (30minRecognition score range: 0–30). During the recognition condition, the 15 previously presented words were presented among 15 distracter words. When taking gender, age, and educational level into account, the total learning score (15WT-total) and the delayed recall score (30minRecall) can be transformed to *T*-scores (Van der Elst et al., 2005). In addition to this standard protocol, we added extended long-term delayed recall (week Recall) and recognition (week Recognition) tests. In the test using the long-term delayed recognition condition, the original 15 distracters were replaced with 15 alternative distracters that were chosen from a parallel 15WT version (matching word frequencies). To prevent rehearsal of the material, subjects were not informed about the 1-week delayed test. The delayed test was presented during a regularly scheduled telephone call conducted either to discuss some of the remaining test results from the neuropsychological assessment or to make a follow-up appointment.

The MMQ was used to measure the following: (1) the degree of concern and contentment regarding memory functioning (MMQ-contentment, 18 items, score range 0–72, Cronbach’s α 0.92), (2) rating of daily forgetfulness (MMQ-ability, 20 items, score range 0–80, Cronbach’s α 0.89), and (3) the frequency of daily memory strategy use (MMQ-strategy, 19 items, score range 0–76, Cronbach’s α 0.86). The MMQ-contentment and MMQ-ability subscales are positively defined, with lower scores indicating less memory contentment and a report of more daily forgetfulness. Higher scores on the MMQ-strategy scale indicate more use of memory strategies than lower scores (Troyer and Rich, 2002; Van der Werf and Vos, 2011).

*T*-tests for independent samples were used to compare the demographic and main outcome variables of both the patient and control groups. To compare initial learning and subsequent forgetting between the patients and controls, a mixed model repeated measures analysis was conducted with the initial five learning trial scores and the subsequent two recall scores (trial 1–5, 30-min recall score, and week recall score).

The aforementioned analyses were also carried out to test whether in the patient group the global outcome of the neuropsychological assessment was related to initial learning and subsequent forgetting, or to the outcomes on the MMQ measures.

Because there were two delayed recognition scores (one after 30 min and another after 1 week) and because the recognition scores from the 15WT are known to be skewed, we calculated the percentage of recognition information loss (% of recognition that was forgotten) as follows: 100^∗^((30minRecognition – week Recognition)/30minRecognition). A similar measure was calculated for the percentage of information loss between the 30-min recall and one-week recall measures (% of recall that was forgotten). These long term information loss measures were used to investigate the relationships (Pearson’s *r*) between forgetting and ratings of subjective memory ability both for the patient and control groups.

## Results

### Participants

Patients were referred by their neurologist or psychiatrist for neuropsychological assessment. The referral questions pertained to the estimation of cognitive function and possible explanations for the cognitive complaints and/or dysfunctions. Thirty-five of the 65 referred patients had received a neurological diagnosis either just prior to the assessment or in their past medical history. The neurological diagnoses varied (e.g., whiplash, traumatic brain injury, obstructive sleep apnea, Parkinson’s disease, multiple sclerosis, stroke) and, for some patients, consisted of multiple diagnoses. The most frequent diagnoses in this group were traumatic brain injury (*N* = 10) and stroke (*N* = 8).

The remaining group of 30 patients had no formal neurological diagnosis. Six of these patients were referred because of possible dementia. The remaining 24 patients reported concerns about and/or impairments in their cognitive functioning. In all of these patients, comorbid somatic conditions (e.g., fibromyalgia, migraine, Crohn’s disease) or psychological distress (e.g., depression, burn-out, anxiety) were possible secondary causes for the cognitive complaints. In total, 63 of the 65 patients were retested after a week’s interval. According to the outcomes of the clinical neuropsychological assessments 35 patients (53.8%) had no cognitive dysfunction, 24 patients (36.9%) had possible signs of minor cognitive dysfunctions, while five patients (7.8%) had definite signs of cognitive dysfunctions. The neuropsychological report of one patient (1.5%) could not be traced.

### Demographics and Standard Memory Test Results

Between the control and patient groups, there was no significant difference in the mean age [*t*(104) = -0.34, *p* = 0.73, *d* = -0.06] or in the mean educational level [*t*(104) = -0.24, *p* = 0.81, *d* = -0.05]. However, the patient group was less content [*t*(102) = -8.18, *p* < 0.01, *d* = -1.62] and reported more daily forgetfulness [*t*(73) = -5.72, *p* < 0.01, *d* = -1.51] than the control group. The patient group also reported significantly more use of memory strategies than the controls [*t*(102) = 2.46, *p* = 0.02, *d* = 0.49]. The standardized measures from the 15WT-total score and the 30minRecall score did not differ significantly between the patient and control groups [standardized 15WT-total: *t*(104) = -0.72, *p* = 0.47, *d* = -0.14; standardized 30minRecall score: *t*(104) = -1.46, *p* = 0.15, *d* = -0.29]. In both the control and patient groups, the mean standardized scores approached that of the normative means (*T*-score = 50) (**Table 1**).

**Table 1:** Demographic data and mean scores and standard deviations of the 15 words test, forgetting rates and multimodal memory questionnaire.

	Patients (baseline *N* = 65, follow-up *N* = 61)			Controls (*N* = 41)	
Demograhpics	Mean (*SD*)	Range		Mean (*SD*)	Range
Number of males: females	35: 30			21: 20	
Age (Years)	51.9 (10.5)	31–76		52.6 (10.1)	36–82
Education level^1^	5.3 (1.4)	1–7		5.4 (1.7)	1–7
**Results 15 words list learning**				
15WT 5th trial	12.0 (2.0)	8–15		12.1 (2.1)	7–15
15WT-total score	47.2 (8.8)	32–66		48.9 (8.3)	25–65
15WT-total *T*-score	50.0 (9.0)	36–67		51.2 (7.0)	37–68
30minRecall	10.0 (2.3)	5–15		10.6 (2.4)	5–15
30minRecall T-score	49.7 (8.2)	36–69		52.0 (7.1)	38–68
30minRecognition	29.0 (1.2)	25–30		29.4 (1.0)	27–30
Week Recall	3.8 (3.1)	0–13	^∗^	5.9 (2.1)	0–12
Week Recognition	25.6 (3.2)	18–30	^∗^2^^	27.0 (1.9)	23–30
% Loss recall	64.0 (25.1)	9–100	^∗^	42.6 (19.2)	-25–82
% Loss recognition	11.9 (9.2)	0–32	^∗^	8.0 (5.7)	0–20
**Results subjective memory ability**					
MMQ-contentment	31.8 (13.1)	5–66	^∗^	51.8 (10.6)	28–71
MMQ-ability	47.0 (12.0)	15–67	^∗^	59.0 (7.7)	42–75
MMQ strategy	30.2 (12.9)	3–54	^∗^	24.5 (8.9)	6–44

*^∗^refer to significant findings: *p* < 0.05. ^1^Educational level refers to the Verhaege classification (range 1–7) of the Dutch education system. The lowest score corresponds to less than 6 years of education (unfinished elementary school), while the highest score (7) represents a university master degree (at least 18 years of education). ^2^Non-parametric testing. 15WT-total score is the total number of words recalled over sessions 1–5 of the Dutch 15 words test. 15WT-total *T*-score refers to an age and education adjusted standardized score of the 15WT-total score. 30 min Recall is the number of words recalled after thirty minutes, and 30 min Recall *T*-score refers to an age and education adjusted standardized score of this measure (Van der Elst et al., 2005). Week Recall is the: number of words recalled after 1 week. 30 min Recognition is the number of correctly identified words out of al list 30 words (15 targets + 15 distracters). Week Recognition is the number of correctly identified words out of al list 30 (15 targets + 15 distracters). % loss recall is the percentage of the 30 min Recall that was forgotten after a 1-week period. % loss recognition is the percentage of the 30 min Recognition that was forgotten over an 1-week period MMQ-contentment, MMQ- ability and MMQ-strategy refer to the mean scores of the three subscales of the multifactorial memory questionnaire.*

### Forgetting

**FIGURE 1:**
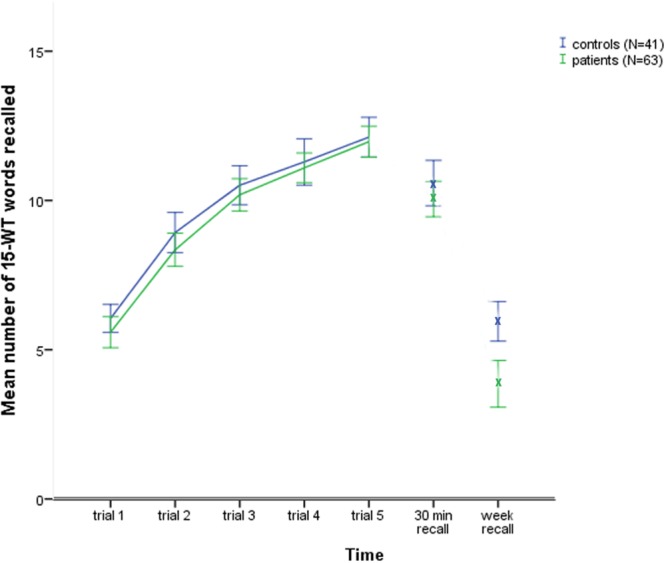
**The control and patients groups 15 words test learning curves and subsequent delayed recall scores**.

A General Linear Model repeated measures analysis was conducted with the factors of group (patients and controls) and time (5 subsequent learning trials, 30minRecall and week Recall). The Mauchly’s test results indicated that the assumption of sphericity had been violated [χ^2^(20) = 102.69, *p* < 0.01]; therefore, the degrees of freedom were corrected using the Greenhouse–Geisser estimates of sphericity (𝜀 = 0.71). The results showed a non-significant main effect for group [*F*(1,102) = 3.12, *p* = 0.08, $\eta_{p}^{2}$ = 0.03]. However, there was a significant main effect for time [*F*(4.3,437.1) = 328.0, *p* < 0.01, $\eta_{p}^{2}$ = 0.76] and a significant interaction of group × time [*F*(4.3,437.1) = 4.94, *p* < 0.01, $\eta_{p}^{2}$ = 0.05]. The plot in **Figure 1** shows the greatest difference between the groups for the measure of week Recall. Subsequent testing indicated that both groups differed significantly only for the 1-week delayed recall test [*t*(102) = -3.78, *p* < 0.01, *d* = -0.74].

After 1 week, the patients lost 64.0% of the information recalled compared to their 30minRecall score, while the controls lost 42.6% of the recalled information [*t*(102) = 4.65, *p* < 0.01, *d* = -0.92].

The percentage of recognition that was forgotten with time differed significantly for both groups [*t*(102) = 2.47, *p* = 0.02, *d* = -0.49]. The recognition scores showed a mean decline in the patient group of 11.9% and a mean decline in the control group of 8.0% over 1 week (**Table 1**).

In order to test whether the outcome of neuropsychological assessment was related to initial learning and subsequent forgetting a General Linear Model repeated measures analysis was conducted with the factors of group (no cognitive dysfunction, possible minor cognitive dysfunction, definite cognitive dysfunction) and time (5 subsequent learning trials, 30minRecall and week Recall). The Mauchly’s test results indicated that the assumption of sphericity had been violated [χ^2^(20) = 90.95, *p* < 0.01]; therefore, the degrees of freedom were corrected using the Greenhouse–Geisser estimates of sphericity (𝜀 = 0.60). The results showed a non-significant interaction of group × time [*F*(7.2,214.1) = 0.25, *p* = 0.97, $\eta_{p}^{2}$ = 0.008]. The main effect for group was non-significant but showed a trend [*F*(1,102) = 3.12, *p* = 0.08, $\eta_{p}^{2}$ = 0.03]. There was a significant main effect for time [*F*(3.6,214.1) = 124.7, *p* < 0.01, $\eta_{p}^{2}$ = 0.68].

The results of the one-way ANOVA’s indicated that there were no significant effects of outcome of neuropsychological assessment on levels of: MMQ-contentment: *F*(2,61) = 0.56, *p* = 0.58; MMQ-ability: *F*(2,61) = 1.40, *p* = 0.25; MMQ-strategy: *F*(2,59) = 0.53, *p* = 0.59; Percentage loss recall: *F*(2,59) = 0.43, *p* = 0.65; Percentage loss recognition: *F*(2,61) = 1.05, *p* = 0.36.

### Relationship between Memory Ratings and Verbal Memory Performance

The correlations between the MMQ variables and memory measures are shown in **Table 2**. In the patient group, the MMQ-contentment and MMQ-ability ratings were significantly and positively correlated with the standardized 15WT-total score and higher recognition scores after 1 week. In addition, the MMQ-ability rating was significantly and positively correlated with the standardized 30minRecall score. None of the MMQ ratings in the patient group were significantly related to the percentage of either recall or recognition that was forgotten after 1 week.

**Table 2:** Pearson’s correlations between the memory performance measures and the subjective memory ability measures.

	Patients (*N* = 65)			Controls (*N* = 41)		
	MMQ-contentment	MMQ-ability	MMQ-strategy	MMQ-contentment	MMQ-ability	MMQ-strategy
15WT-total *T*-score	0.30 (*p* = 0.014)	0.39 (*p* = 0.002)	-0.07 (*p* = 0.586)	-0.02 (*p* = 0.897)	-0.03 (*p* = 0.856)	-0.00 (*p* = 0.974)
30minRecall *T*-score	0.21 (*p* = 0.094)	0.30 (*p* = 0.015)	-0.18 (*p* = 0.159)	-0.07 (*p* = 0.658)	0.11 (*p* = 0.497)	-0.15 (*p* = 0.355)
Week recall	0.14 (*p* = 0.270)	0.16 (*p* = 0.200)	-0.01 (*p* = 0.959)	-0.05 (*p* = 0.766)	0.10 (*p* = 0.537)	0.25 (*p* = 0.114)
% loss recall	-0.10 (*p* = 0.447)	-0.08 (*p* = 0.522)	0.02 (*p* = 0.888)	-0.00 (*p* = 0.990)	-0.12 (*p* = 0.461)	-0.25 (*p* = 0.107)
Week recognition	0.26 (*p* = 0.041)	0.33 (*p* = 0.010)	-0.10 (0.458)	0.20 (*p* = 0.207)	0.24 (*p* = 0.133)	-0.02 (*p* = 0.883)
% loss recognition	-0.22 (*p* = 0.089)	-0.23 (*p* = 0.070)	0.05 (*p* = 0.723)	-0.15 (*p* = 0.347)	-0.26 (*p* = 0.100)	0.13 (*p* = 0.398)

*Data analyses pertain to the groups of patients and controls with both baseline and follow-up data. 15WT-total *T*-score refers to an age and education adjusted standardized score of the total number of words recalled over sessions 1–5 of the Dutch 15 words test. 30 min Recall *T*-score refers to an age and education adjusted standardized score of this measure (Van der Elst et al., 2005). % loss recall is the percentage of the 30 min Recall that was forgotten after a 1-week period. % loss recognition is the percentage of the 30minRecognition that was forgotten over an 1-week period. MMQ-contentment, MMQ- ability and MMQ-strategy refer to the mean scores of the three subscales of the multifactorial memory questionnaire.*

In the control group, no significant relationships were found between the MMQ ratings and any of the memory performance measures.

Since there were trends with the long term percentage loss measures, Fisher’s *r*- to –*z* transformations were carried out to examine whether the correlations with the standard memory measures were any stronger compared to those at 1 week. We choose to contrast he highest association that was found with standard memory measure (MMQ-ability with standardized 15WT-total score, Pearson’s *r* = 0.39) to the highest correlation between MMQ-ability and the percentage loss measures (Pearson’s *r* = -0.23), while taking into account that the negative direction of these correlation due to the definition of percentage loss. The outcome (*Z* = 0.861, *p* = 0.195) indicated that both correlations did not differ significantly from each other.

We reasoned *ad hoc* that if anosognosia would be an issue, this most likely would be the case in the five patients who showed definite signs of cognitive impairment at neuropsychological assessment. Therefore, the aforementioned analyses were repeated while excluding these patients. Again, a significant interaction effect [*F*(4.3,409.2) = 4.89, *p* < 0.01, $\eta_{p}^{2}$ = 0.05] was found between group (patients and controls) and time (initial learning and subsequent forgetting). In this patient group the associations between subjective memory ability and memory scores altered slightly, with only the correlations between MMQ-contentment and standardized 15WT-total score (*r* = 0.28, *p* = 0.032), MMQ-ability and standardized 15WT-total score (*r* = 0.41, *p* = 0.001), and MMQ-ability and standardized 30minRecall score (*r* = 0.31, *p* = 0.019), reaching significance.

## Discussion

The results of this study did not confirm our expectation that long term forgetting rates would explain subjective memory ability better than the standardly used immediate and delayed recall scores. In our clinical group the associations between subjective memory ability and memory performance indices were small (R2 ∼ 15%) and resembled the small effect sizes that have been reported in various studies that assessed relations between subjective and objective memory performances in older adults (Jungwirth et al., 2004; Crumley et al., 2014). Although the patients showed somewhat steeper forgetting rates over a week’s period, both subjective memory ability and the amount of long term forgetting were not related to the presence of cognitive dysfunction. In this study the classification of cognitive dysfunction was not based on objective and specific criteria such as scores at a clinical severity scale or tests of general efficiency. Instead the classification was based on the conclusion paragraphs of the clinical neuropsychological reports and might therefore have been subject to variation. However, compared to screening instruments such as the Expanded Disability Status Scale or Montreal Cognitive Assessment, a comprehensive neuropsychological assessment can be regarded as more sensitive to detect mild cognitive deterioration or signs of poor effort.

One could speculate that the higher long term forgetting rates and lower subjective ability in the patient group, and their small but significant associations, might be evidence for the existence of subtle brain or cognitive dysfunction affecting long term consolidation. Some studies have indeed suggested that memory complaints may be associated with changes in the brain before an actual decline in performance on standard memory tests is observed. Saykin et al. (2006) showed for example that a group of older adults with cognitive complaints but normal neuropsychological test-performance had gray matter atrophy patterns comparable to that of patients with amnestic mild cognitive impairment. Wang et al. (2012) used diffusion tensor imaging to demonstrate parahippocampal white matter changes in a similar group of patients with cognitive complaints. However, in our study it is difficult to explain the increased long term forgetting rates as a sign of subtle cognitive dysfunction association. The trend of the negative impact of cognitive dysfunction was not restricted to long term forgetting but affected both initial learning as well as the subsequent retrieval stages. This seems somehow in contrast to the line of thought that subtle factors might have influenced consolidation specifically. Moreover, when we compare our findings to long term forgetting rates in healthy subjects, the recall forgetting percentages appear to fall within the range that was reported in the verbal list learning study of Slamecka and McElree (1983). They studied normal forgetting and the influence of initial learning and found that the subjects would forget almost half of the initially presented information within approximately 5 days. As such, one could argue that the found differences in forgetting rates do not reflect impaired consolidation in the patient group but might possibly reflect normal forgetting and can be explained by methodological factors such as contextual differences during testing or the heterogeneous background of our patient group.

In both our patient and control groups the long-term recognition forgetting rates were small. This indicates that most of the initially learned material was well encoded and consolidated. The effect size of the differences between patients and controls was more than double for the long term retrieval measure compared to the long term recognition measure. The patients seemed to have relatively more difficulties with the long term retrieval of previously learned material. This finding underlines the importance of using both recall and recognition procedures, as was recommended in both reviews of accelerated forgetting in clinical patients groups (Elliott et al., 2014; Geurts et al., 2015). In clinical practice, poor recall has been associated with poor encoding and retrieving of the information. Poor recognition has been associated with mainly encoding of newly acquired information. The better the information has been encoded, the better the recognition is (Neath and Surprenant, 2003). Although theoretical memory models, such as the Search of Associative Memory (SAM) model, predict that a delay decreases both recall and that retrieval processes, retrieval processes are more prone to interference through changes in retrieval and contextual cues (Gillund and Shiffrin, 1984). It is possible that our patient group was subjected to more interference since the initial verbal memory testing was part of a complete clinical neuropsychological assessment while the controls had only a limited memory assessment. Another possible bias might be the context in which the 1 week assessment took place. All of the control subjects were contacted by telephone, while the majority of patients were retested at the location of baseline testing. These contextual cue differences might explain the larger effect size of the long term recall forgetting difference between both groups. It is possible that the control group would have had better week recall when asked in the same baseline testing location. It is possible that in our patient group the differences in recall context (e.g., testing by telephone or at baseline location) might have introduced noise. This was, however, not confirmed when we *post hoc* restricted our analyses only to patients who were tested at the baseline location. Similar patterns of associations between subjective memory ability and long term forgetting were found as in the total patient group.

An alternative behavioral explanation for our findings might be found within the heterogeneous background of our patient group. A substantial number of our patients were referred either to rule out cognitive decline in the absence of neurological or radiological abnormalities, or to differentiate between possible causal factors for their cognitive complaints (e.g., comorbid psychiatric symptoms). Perhaps because these subjects were concerned about their memory function, they were more acutely aware of it and were therefore better able to judge their daily forgetfulness. Negative subjective memory ability ratings may also reflect poor performance expectations that are sometimes observed in clinical practice when patients make little effort during recall sessions. Such factors may also increase proneness to interference and contribute to both forgetting and the self-report of poor memory ability. These sources of interference perhaps do not impact the relatively short clinical testing sessions but may accumulate over time and cause an increase in memory complaints and a decrease in long-term recall. However, we did not relate our forgetting percentages to other possible comorbid variables, such as depressive symptoms or sleep complaints. Another limitation of this study was that the findings and conclusions were limited to only one type of long-term delayed memory—namely, the consolidation of a previously learned list of words. It could be argued that subjective memory ability ratings reflect consolidation or retrieval difficulties of more complex and associated verbal content, or visual information, or perhaps previously implicitly learned material. Future assessments should therefore involve multiple multi-domain memory measures.

## Conclusion

The suggestion of patients that standard memory test intervals are too short to capture their perceived memory problems could not be affirmed by the findings of our study. No evidence was found that subjective memory ability ratings were more strongly related to the long term forgetting indices compared to the standard memory test scores. The presence of cognitive dysfunctions, other than memory dysfunction, was not related to subjective memory ability and did not selectively affect long term forgetting. As such, long term forgetting rates did not seem to be a more sensitive measure for perceived memory ability than the standard used memory test scores. Therefore, and for practical reasons, we do not recommend implementing long term memory intervals as standard practice in clinical neuropsychological assessment.

## Author Contributions

SW initiated this study, collected and analyzed the data and participated in writing the article. SG collected data and participated in data analysis and writing the article. MW collected data and participated in writing the article.

## Conflict of Interest Statement

The authors declare that the research was conducted in the absence of any commercial or financial relationships that could be construed as a potential conflict of interest.

## References

[B1] Aben L. Ponds R. W. H. M. Heijenbrok-Kal M. H. Visser M. M. Busschback J. J. V. Ribbers G. M. (2011). Memory complaints in chronic stroke patients are predicted by memory self-efficacy rather than memory capacity. *Cerebrovasc. Dis.* 31 566–572. 10.1159/000324627 21487221

[B2] Butler C. R. Zeman A. Z. (2008). Recent insights into the impairment of memory in epilepsy: transient epileptic amnesia, accelerated long-term forgetting and remote memory impairment. *Brain* 131 2243–2263. 10.1093/brain/awn127 18669495

[B3] Carlesimo G. A. Sabbadini M. Fadda L. Caltagirone C. (1995). Forgetting from long-term memory in dementia and pure amnesia: role of task delay of assessment and aetiology of cerebral damage. *Cortex* 31 285–300. 10.1016/S0010-9452(13)80363-2 7555007

[B4] Crumley J. J. Stetler C. A. Horhota M. (2014). Examining the relationship between aubjective and objective memory performance in older adults: a Meta-Analysis. *Psychol. Aging* 29 250–263. 10.1037/a0035908 24955993

[B5] DeLuca J. Gaudino E. A. Diamond B. J. Christodoulou C. Engel R. A. (1998). Acquisition and storage deficits in multiple sclerosis. *J. Clin. Exp. Neuropsychol.* 20 376–390. 10.1076/jcen.20.3.376.819 9845164

[B6] Elliott G. Isaac C. L. Muhlert N. (2014). Measuring forgetting: a critical review of accelerated long-term forgetting studies. *Cortex* 54 16–32. 10.1016/j.cortex.2014.02.001 24631847 PMC4007031

[B7] Gaudino E. A. Chiaravalloti N. D. DeLuca J. Diamond B. J. (2001). A comparison of memory performances in relapsing-remitting, primary progressive and secondary progressive, multiple sclerosis. *Neuropsychiatry Neuropsychol. Behav. Neurol.* 14 32–44. 11234907

[B8] Geurts S. van der Werf S. P. Kessels R. P. C. (2015). Accelerated forgetting? An evaluation on the use of long-term forgetting rates in patients with memory problems. *Front. Psychol.* 6:752 10.3389/fpsyg.2015.00752 PMC4460323 26106343

[B9] Gillund G. Shiffrin R. M. (1984). A retrieval model for both recognition and recall. *Psychol. Rev.* 91 1–67. 10.1037/0033-295X.91.1.1 6571421

[B10] Gold P. E. (2006). The many faces of amnesia. *Learn. Mem.* 13 506–514. 10.1101/lm.277406 17015847

[B11] Jungwirth S. Fischer P. Weissgram S. Kirchmeyr W. Bauer P. Tragl K. (2004). Subjective memory complaints and objective memory impairment in the Vienna-Transdanube aging community. *J. Am. Geriatr. Soc.* 52 263–268. 10.1111/j.1532-5415.2004.52066.x 14728638

[B12] Manes F. Serrano C. Calcagno M. L. Cardozo J. Hodges J. (2008). Accelerated forgetting in subjects with memory complaints. A new form of mild cognitive impairment? *J. Neurol* 255 1067–1070. 10.1007/s00415-008-0850-6 18484236

[B13] McGaugh J. L. (2000). Memory: a century of consolidation. *Science* 287 248–251. 10.1126/science.287.5451.248 10634773

[B14] Neath I. Surprenant A. M. (2003). *Human Memory.* Wadsworth: Thomson.

[B15] Saan R. J. Deelman B. G. (1986). *De Nieuwe 15-Woordentest (A en B). Een Handleiding*. Lisse: Swets & Zeitlinger.

[B16] Saykin A. J. Wishart H. A. Rabin L. A. Santulli R. B. Flashman L. A. West J. D. (2006). Older adults with cognitive complaints show brain atrophy similar to that of amnestic MCI. *Neurology* 12 834–842. 10.1212/01.wnl.0000234032.77541.a2 16966547 PMC3488276

[B17] Slamecka N. J. McElree B. (1983). Normal forgetting of verbal lists as a function of their degree of learning. *J. Exp. Psychol. Learn. Mem. Cogn.* 9 384–397.

[B18] Squire L. R. Alvarez P. (1995). Retrograde amnesia and memory consolidation: a neurobiological perspective[review]. *Curr. Opin. Neurobiol.* 5 169–177. 10.1016/0959-4388(95)80023-9 7620304

[B19] Troyer A. K. Rich J. B. (2002). Psychometric properties of a new metamemory questionnaire for older adults. *J. Gerontol.* 57B 19–27. 10.1093/geronb/57.1.P19 11773220

[B20] Van der Elst W. van Boxtel M. P. J. van Breukelen J. P. Jolles J. (2005). Rey’s verbal learning test: normative data for 1855 healthy participants aged 24-81 years and the influence of age, sex, education, and mode of presentation. *J. Int. Neuropsychol. Soc.* 11 290–302. 10.1017/S1355617705050344 15892905

[B21] Van der Werf S. P. Vos S. H. (2011). Memory worries and self-reported daily forgetfulness: a psychometric evaluation of the Dutch translation of the multifactorial memory questionnaire. *Clin. Neuropsychol.* 25 244–268. 10.1080/13854046.2010.543290 21253959

[B22] Vestberg S. Passant U. Risberg J. Elfgren C. (2007). Personality characteristics and affective status related to cognitive test performance and gender in patients with memory complaints. *J. Int. Neuropsychol. Soc.* 13 911–919. 10.1017/S1355617707071159 17942009

[B23] Walsh C. M. Wilkins S. Bettcher B. M. Butler C. R. Miller B. L. Kramer J. H. (2014). Memory consolidation in aging and MCI after 1 week. *Neuropsychology* 28 273–280. 10.1037/neu0000013 24219610 PMC4211844

[B24] Wang Y. West J. D. Flashman L. A. Wishart H. A. Santulli R. B. Rabin L. A. (2012). Selective changes in white matter integrity in mCI and older adults with cognitive complaints. *Acta Biochem. Biophys.* 1822 423–430. 10.1016/j.bbadis.2011.08.002 PMC3235544 21867750

